# Alterations in *ZENK* and glucagon RNA transcript expression during increased ocular growth in chickens

**Published:** 2010-04-13

**Authors:** Regan Ashby, Peter Kozulin, Pam L. Megaw, Ian G. Morgan

**Affiliations:** 1 Research School of Biology, Australian National University, Canberra, Australia; 2 ARC Centre of Excellence in Vision Science, Australian National University, Canberra, Australia; 3 Department of Physiology and Pharmacology, School of Veterinary and Biomedical Sciences, James Cook University, Townsville, Australia

## Abstract

**Purpose:**

To examine in detail the time-course of changes in *Zif268, Egr-1, NGFI-A, and Krox-24* (*ZENK*) and pre-proglucagon (*PPG*) RNA transcript levels in the chick retina during periods of increased ocular growth induced by form-deprivation and negative-lens wear. To further elucidate the role of *ZENK* in the modulation of ocular growth, we investigated the effect of intravitreal injections of the muscarinic antagonist atropine and the dopamine agonist 2-amino-6,7-dihydroxy-1,2,3,4-tetrahydronaphthalene hydrobromide (ADTN), both of which block the development of experimental myopia, on the expression of *ZENK* in eyes fitted with negative-lenses.

**Methods:**

Myopia was induced by fitting translucent diffusers or −10D polymethyl methacrylate (PMMA) lenses over one eye of the chicken. At times from 1 h to 10 days after fitting of the diffusers or negative lenses, retinal RNA transcript levels of the selected genes were determined by semi-quantitative real-time reverse transcriptase polymerase chain reaction (RT–PCR). For the pharmacology experiments, −10D lenses were fitted over the left eye of chicks for a period of 1h. Intravitreal injections of atropine (10 μl–25 mM), ADTN (10 μl–10 mM), or a vehicle solution were made immediately before fitting of the lenses.

**Results:**

*ZENK* RNA transcript levels were rapidly and persistently down-regulated following the attachment of the optical devices over the eye. With a delay relative to *ZENK*, *PPG* transcript levels were also down-regulated. Induced changes in gene expression were similar for both form-deprivation and negative-lens wear. When atropine or ADTN were administered immediately before lens attachment, the rapid down-regulation in *ZENK* RNA transcript levels normally seen following 1 h of negative-lens wear was not seen, and *ZENK* transcript levels rose above those values seen in control eyes. However, injection of atropine or ADTN into untreated eyes had no effect on *ZENK* transcript levels.

**Conclusions:**

Both form-deprivation and negative-lens wear modulated the retinal expression of *ZENK* and *PPG* RNA transcripts, with a similar time-course and strength of response. The ability of the tested drugs to prevent the down-regulation of *ZENK* in both lens-induced myopia (LIM) and form-deprivation myopia (FDM) suggests that atropine and ADTN act directly and rapidly on retinal circuits to enhance sensitivity early in the signaling process. These findings suggest that very similar molecular pathways are involved in the changes in eye growth in response to form-deprivation and negative lenses at 1 h after the fitting of optical devices.

Received: January 22, 2010

Accepted: April 6, 2010

## Introduction

The emergence of a myopia epidemic in urban East Asia (for review see Morgan and Rose [1]), has prompted considerable research to understand the molecular pathways involved in the regulation of ocular growth (for review see [2]). It is generally believed that the pathways involved in the control of eye growth involve signal cascades initiated in the retina, which send signals through the retinal pigment epithelium (RPE) and choroid to control the growth of the sclera. However, the identities of the retinal molecules and pathways involved are still unclear. Recent work has investigated global changes in retinal gene expression during the development of experimental myopia [3-8], giving some insight into possible molecules involved in the regulation of ocular growth.

Two important candidate molecules are the retinal peptides, Zif268, Egr-1, NGFI-A, or Krox-24 (ZENK) and glucagon, which have previously been implicated in the control of eye growth [3,9-11]. *ZENK*, is a member of the immediate early gene (IEG) family of transcriptional regulators, and is the avian ortholog of the IEG *Egr-1*. It encodes for a short-lived nuclear protein with a zinc finger binding domain. Its expression is normally rapidly and transiently induced by extracellular stimuli. ZENK has been implicated in the modulation of eye growth due to the observation that the percentage of glucagon-immunoreactive amacrine cells positively labeled for the ZENK peptide shows a bi-directional response to opposing growth stimuli [9]. Following 30 min or 2 h of form-deprivation or negative-lens wear, both of which induce an increase in the rate of axial elongation, the percentage of glucagonergic amacrine cells positively labeled for ZENK was significantly reduced. In contrast, 2 h of either positive-lens wear or removal of diffusers from previously form-deprived eyes, both of which reduce the rate of eye growth, caused a significant increase in the percentage of glucagonergic amacrine cells positively labeled for the ZENK peptide. Schippert et al. [12] have reported a relative myopic shift in *Egr-1* knockout mice as compared to wild-type control animals, further supporting a role for this IEG in growth modulation. At the RNA transcript level, Simon et al. [13] have reported significant down-regulation in *ZENK* levels following 30 min and 2 h of negative-lens wear. The molecular studies have therefore only examined relatively short exposure times, whereas modulation of eye growth is prolonged.

Glucagon is a 29-amino-acid long peptide produced by the proteolytic cleavage of the precursor molecule pre-proglucagon (PPG) [14]. Glucagon is part of a superfamily of secretin-glucagon peptides that act through G-protein coupled receptors, and has increasingly been identified as a possible neurotransmitter in the central nervous system [15,16]. Exposure to negative lenses has been shown to increase glucagon peptide levels, as measured by radioimmunoassay, after 24 h but not after 4 h of lens wear [10]. At the RNA transcript level, Buck et al. [11] reported that *PPG* levels were initially upregulated following 2 h of negative-lens treatment, before showing significant down-regulation after 24 h of lens wear. It has also been demonstrated that glucagon agonists can prevent experimentally induced myopia [17,18], while glucagon antagonists can prevent compensation for positive-lens wear [18], suggesting a role for glucagon in the modulation of eye growth.

To gain more detailed information on the coupling of changes in *ZENK* and *PPG* RNA transcript levels with changes in the rate of ocular growth stimulated by visual manipulation, we studied the changes in transcript levels at times from 1 h to 10 days, using the two paradigms that promote axial elongation–form-deprivation myopia (FDM) and lens-induced myopia (LIM). To analyze whether the eye uses similar mechanisms in FDM and LIM, we compared the changes in gene expression seen during the development of FDM and LIM, and also compared the effects of atropine and ADTN, both capable of retarding development of experimental myopia in chicks [19], on early changes in *ZENK* expression in the two paradigms.

## Methods

### Animal housing

One-day-old male Australorp chickens were obtained from Barter and Sons Hatchery, Luddenham, NSW, Australia. Chickens were maintained in temperature-controlled rooms under a 12:12 h light–dark cycle, with incandescent illumination of ~500 lux during the light phase, and <1 lux in the dark phase (lights on at 6 am and off at 6 pm). Chickens had access to unlimited amounts of food and water, and were given three days to become accustomed to their surroundings before experiments were started. All experiments were approved by the Australian National University Animal Experimentation Ethics Committee (Protocols R.VS.14.03 and R.VS.18.05) and conformed to the ARVO Resolution on the Use of Animals in Ophthalmic and Vision Research.

### Experimental treatment – visual manipulation

The methods used for induction of FDM, which results from increased axial elongation of the eye, have been previously described in detail [6,8]. Briefly, FDM was induced monocularly by fitting a translucent diffuser over the left eye of 5-day-old chickens, using Velcro^®^ mounts. The diffusers were made from Wellcome Codral^®^ (Johnson and Johnson Pacific Pty Ltd, Sydney, Australia) blister strips, and reduced light intensity by ~0.4 log units. LIM, which also results from increased axial elongation of the eye, was induced by fitting −10D PMMA lenses over the left eye of five-day-old chickens, using similar Velcro^®^ mounts.

For the analysis of changes in gene expression during the development of FDM (n=90) or LIM (n=90), five-day-old chickens were exposed to the diffusers or negative lenses for periods of 1 h or 1, 3, 7, or 10 days. For both FDM and LIM, nine samples per experimental condition were collected at each time point. Each sample contained two retinas from experimental eyes, one from each of two animals. Retinas from contralateral eyes were also collected, and processed separately from internal contralateral controls. In parallel, pooled retinas were collected from age-matched untreated control animals (n=9) at each time point for all experimental conditions.

### Intravitreal injection protocol

Chickens were split into eight groups: (1) injection of an atropine solution immediately before fitting a negative lens (n=6), (2) injection of an atropine vehicle solution (distilled water) immediately before fitting a negative lens (n=6), (3) injection of an 2-amino-6,7–18 dihydroxy-1,2,3,4-tetrahydronaphthalene hydrobromide (ADTN) solution immediately before fitting a negative lens (n=6), (4) injection of an ADTN vehicle solution (1% [w/v] ascorbic acid) immediately before fitting a negative lens (n=6), (5) no injection immediately before fitting a negative lens (injection control, n=6), (6) injection of an atropine solution into otherwise untreated eyes (n=6), (7) injection of an ADTN solution into otherwise untreated eyes (n=6), and (8) age-matched untreated control birds (n=6).

Negative 10D polymethyl methacrylate (PMMA) contact lenses were fitted over the left eyes of chickens at 11 AM on the day of treatment, using the methods described above. In these experiments, lenses were attached for a period of 1 h before the animals were euthanized and retinal tissue collected.

Intravitreal injections into the lens-treated eyes were performed immediately before the attachment of the lenses under light isoflurane anesthesia (5% in 1 l of medical grade oxygen per minute), using a handheld 0.3 ml BD Ultra-Fine II syringe with a 8 mm long 31 gauge needle. The intraocular injections consisted of 10 μl of either 25 mM atropine (Sigma-Aldrich, Castle Hill, NSW, Australia) dissolved in distilled water (pH 7.4), 10 mM 2-amino-6,7-dihydroxy-1,2,3,4-tetrahydronaphthalene hydrobromide (ADTN; Sigma-Aldrich) dissolved in 1% ascorbic acid (pH 7.4), or distilled water or 1% ascorbic acid as vehicles (pH 7.4), as appropriate. Parallel experiments were performed on control birds that were not exposed to any visual manipulation. One injection per eye was made into the vitreous chamber through the sclera in the superior-temporal corner of the eye, by pulling back the eyelid and using an 8 mm long needle to inject into the middle of the vitreous body, so that contact with the retina was avoided. Due to the length of the needle and the angle of insertion, it was not possible to strike the retina; therefore, we did not monitor this process. However, when undertaking dissections of each eye, no indication was seen that any of the retina’s had been punched by a needle.

### Tissue preparation

Chickens were euthanized with CO_2_. Each eye was removed and hemisected equatorially, with the anterior portion of the eye and vitreous body discarded. The posterior eye cup was floated in chilled phosphate-buffered saline (137 mM NaCl, 2.7 mM KCl, 11.3 mM Na_2_HPO_4_, 1.5 mM KH_2_PO_4_, pH 7.4), allowing collection of the retina free of RPE, choroidal, and scleral tissue. Tissue was immediately frozen on dry ice and then stored at −80 °C until use.

Preliminary experiments (results not shown) demonstrated that there were no significant diurnal rhythms in the expression of *ZENK* or *PPG* RNA transcripts sampled at 4 h intervals over a 24 h period, whereas rhythms were detected in retinal clock gene period 2 (*Per2*) RNA transcript expression, similar to those previously reported [20]. This suggests that the time of collection of samples was not an important factor, but, as an additional precaution, all samples were collected between 12 noon and 2 PM

### Preparation of RNA and reverse transcription to cDNA

The methods used for the preparation of RNA, reverse transcription, and quantification by real-time reverse transcriptase (RT)–PCR using the combined Trizol®/Qiagen RNeasy method have been previously described in detail [8,21]. Total RNA purity was checked using gel electrophoresis, and quantified using spectrophotometry. Samples (0.5 μg/μl) were reverse-transcribed to first strand cDNA, which was used as a cDNA template for real-time RT–PCR reactions, using Taq DNA polymerase (Promega, Alexandria, NSW, Australia). The primers used for the analysis of chicken *ZENK* and *PPG* RNA transcript expression, as well as β-actin (*Actb*), are shown in Table 1, and were validated through gel electrophoresis and automated sequencing. Primer efficiency (*E*) was determined from the slope of the curve generated through a cDNA dilution series, using the formula E=10^(−1/slope)^ (Table 1).

**Table 1:** Sequences, slope, efficiency (E) and R^2^ correlation of gene-specific primers used for RT–PCR assays.

**Gene product**	**GenBank**	**Primer (5′-3′) size (bp)**	**Slope**	**Efficiency**	**Correlation**	**Product**
*Egr-1*	AF026082	ACTAACTCGTCACATTCGCA	-3.55	1.91	0.99	241
		TGCTGAGACCGAAGCTGCCT				
*PPG*	NM_205260.1	AGCGTCATTCACAAGGCA	-3.76	1.85	0.98	184
		TCAGAATGACGCTTGGAAAT				
*Actb*	NM_205518	TAAGGATCTGTATGCCAACACAGT	-3.48	1.94	0.99	241
		GACAATGGAGGGTCCGGATTCATC				

### Real-time RT–PCR

All reactions were performed on a RotorGene 3000 RT–PCR cycler (Corbett Research, Doncaster, Victoria, Australia). Cycling conditions included an initial denaturing phase of 95 °C for 5 min, followed by 35 cycles of denaturation at 95 °C for 25 s, annealing at 60 °C for 15 s, and extension at 72 °C for 20 s. Specificity of amplification was confirmed through melt curve analysis and gel electrophoresis of PCR products. Repeat takeoff values and sample amplification values obtained by the RotorGene v6.0 software were transferred into a custom-built Microsoft Excel spread sheet. For graphical representation, the relative expression ratio for each gene was determined by formula [22], with values from age-matched untreated animals serving as control values for determination of changes in gene expression within experimental and contralateral control eyes. The expression of target genes was normalized against the reference gene *Actb*, with repeat takeoff values for *Actb* unaffected by experimental treatments.

### Refraction of chickens

Refraction was measured for treated (n=8 per treatment group) and contralateral control eyes (n=8 per treatment group) daily over a ten-day period after fitting the diffusers or lenses. If a diffuser or lens was dislodged or removed by a bird, that animal was removed from the study. Each chick was anaesthetized using isoflurane as described above. The eyelids of the anaesthetized chick were held open using forceps, while the refractive error was measured by retinoscopy (Heine, Beta 200, Brookvale, NSW, Australia) with wide aperture optical trial lenses. The refraction in each meridian was measured. Refractions were corrected for the 33 cm working distance, and expressed as the spherical equivalent.

### Statistical analysis

Results are presented as mean plus or minus standard error of the mean. Student *t*-tests and a multivariate ANOVA (MANOVA) were performed using JMP 7®, (SAS Institute GmbH, Munich, Germany) with the cut-off for significance at the 5% confidence level. All other calculations were performed in Microsoft® Excel 2003 (Microsoft Corporation, Redmond, WA).

For statistical analyses and graphical representation of changes in *ZENK* and *PPG* RNA transcript expression, the mean normalized expression (MNE) of the target genes was calculated separately for each condition (treated, contralateral control, and age-matched untreated control retinal tissue). The MNE was calculated from the efficiency (*E*) of the target genes to the power of its average crossing threshold (CT) value (*E*^CT^ target), divided by the efficiency (*E*) of the reference gene (*Actb*) to the power of its average CT value (*E*^CT^ reference), following the method of Simon et al. [23]. A MANOVA, followed by the student’s unpaired *t*-test with the Bonferroni correction for multiple testing, was used to compare the effects of different treatment regimes on the MNE over time, and group changes at individual time points on *ZENK* and *PPG* transcript levels. A MANOVA with repeat measures design was used to analyze changes in ocular refraction over time for each treatment regime.

## Results

### Changes in refraction

**Figure 1:**
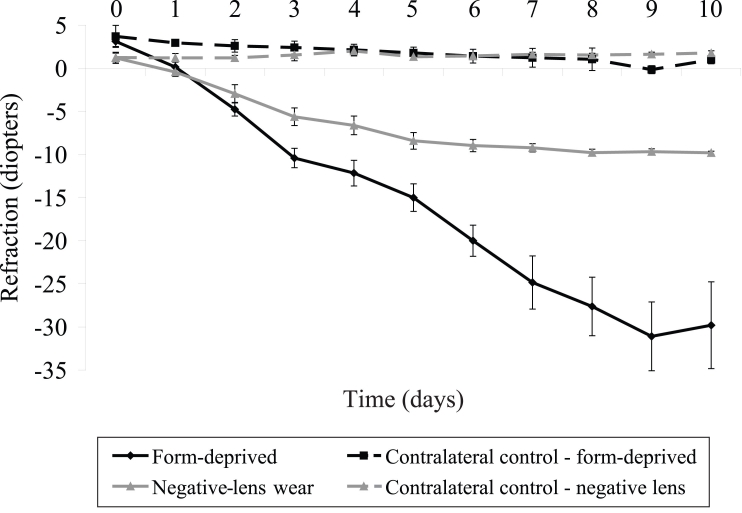
Changes in the refractive error of treated and contralateral control eyes over ten days of form-deprivation and negative-lens wear. The fitment of translucent diffusers over the eye induced significant development of myopia over the ten-day experimental period, as compared to control values (MANOVA; F (1,10)=8.3, p<0.01). Chicks fitted with −10D lenses significantly compensated for the lenses over the initial seven days of treatment (MANOVA; F (1,7)=10.2, p<0.01), before plateauing. Although changes in refraction of contralateral control eyes appeared different between treatment groups, this behavior was not statistically significant over time (MANOVA; F (1,10)=2.04, p=0.18). Error bars represent the standard error of the mean (SEM), n=8 per time, per experimental treatment.

As shown in Figure 1, at baseline, the chickens had on average moderately hyperopic refractions (+3.1±0.6 D), but they rapidly developed FDM, which increased in magnitude over the 10 day experimental period (MANOVA, repeated measures; F (1, 10)=25.8, p<0.0001; Figure 1). In contrast, in the contralateral eyes, the initial moderate hyperopia (+3.7±0.5 D) slowly decreased to a mean refraction close to emmetropia over the experimental period (+0.4±0.2 D; MANOVA, repeated measures; F (1, 10)=8.3, p<0.01).

Chick eyes exposed to −10D lenses compensated over a period of days for the imposed refractive error (Figure 1). Specifically, by the seventh day of negative lens treatment, all eyes had nearly compensated for the imposed hyperopic defocus (−9.2±0.5D). The values plateaued over the remaining three days of refractive measurements (day 8, −9.8±0.4D; day 9, −9.7±0.3D; day 10, −9.8±0.2D). Contralateral control eyes showed little change in refractive error, displaying mild levels of hyperopia over the time-course measured (MANOVA, repeated measures; F (1, 10)=0.34, p=0.84; Figure 1). Although the changes in refraction of contralateral control eyes appeared different between treatment groups (e.g., FDM and LIM), this behavior was not statistically significant over time (MANOVA, repeated measures; F (1, 10)=2.04, p=0.18).

### Changes in *ZENK* RNA transcript expression during development of FDM and LIM

The fitting of translucent diffusers or negative lenses significantly affected *ZENK* RNA transcript levels in the experimental eyes over time, as compared to both contralateral control values (MANOVA; F (2,86)=33.9, p<0.01, and F (2,86)=32.9, p<0.001, respectively) and age-matched untreated control values (MANOVA; F (2,86)=37.9, p<0.001 and F (2,86)=18.5, p<0.01, respectively). There was no significant difference over time in expression of *ZENK* transcript levels between contralateral control eyes, for either the form-deprived or the negative-lens-treated animals and age-matched untreated values (MANOVA; F (2,86)=2.65, p=0.14 and F (2,86)=0.69, p=0.43, respectively).

**Figure 2:**
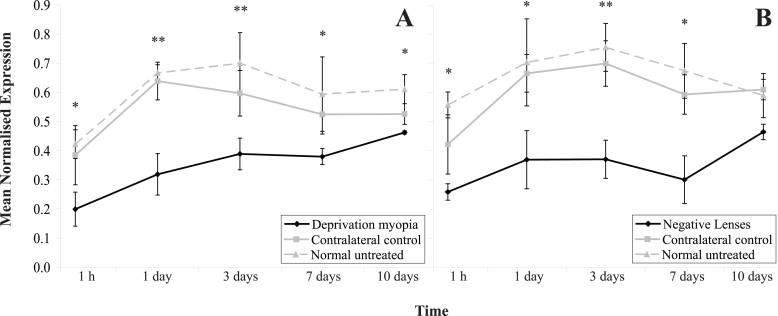
Changes in *ZENK* RNA transcript levels in treated and contralateral control retinas following increased ocular growth induced by the fitting of translucent diffusers or negative lenses. Mean normalized expression of *ZENK* RNA transcript levels from diffuser-treated (**A**) or negative-lens-treated (**B**) eyes following 1 h, 1, 3, 7, and 10 days of treatment. Fitting of translucent diffusers or negative lenses significantly affected *ZENK* RNA transcript levels in the experimental eye over time, as compared to both contralateral control values (MANOVA; F (2,86)=33.9, p<0.01 and F (2,86)=32.9, p<0.001, respectively) and age-matched untreated control values (MANOVA; F (2,86)=37.9, p<0.001 and F (2,86)=18.5, p<0.01, respectively). *ZENK* transcript levels in the contralateral control eyes from either form-deprived or negative-lens-treated animals were unaffected by treatment as compared to age-matched untreated values (MANOVA; F (2,86)=2.65, p=0.14 and F (2,86)=0.69, p=0.43, respectively). The mean normalized expression is calculated from the efficiency (*E*) of the target genes to the power of its average CT value (*E*^CT^, target), divided by the efficiency (*E*) of the reference gene (β-actin) to the power of its average CT value (*E*^CT^, reference). Error bars represent SEM, n=9. (* p<0.05, ** p<0.01).

**Figure 3:**
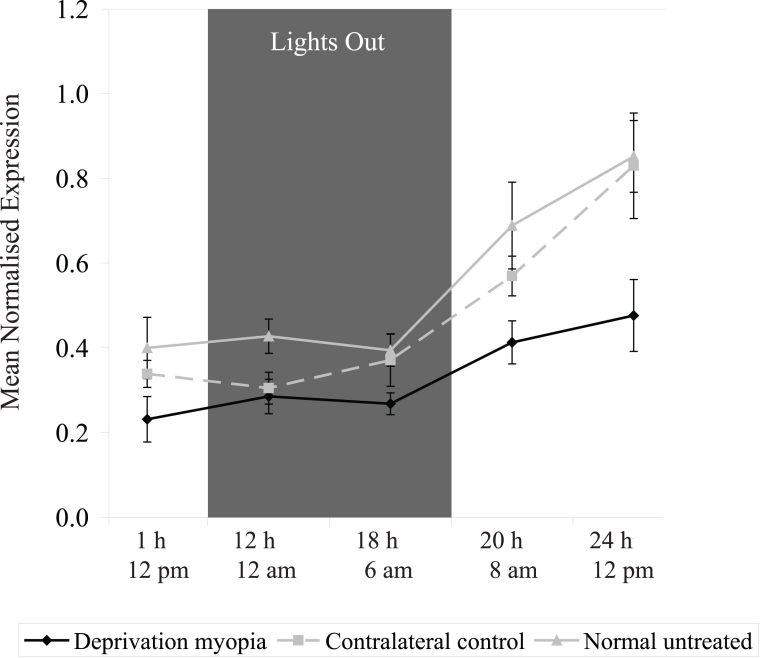
Changes in *ZENK* RNA transcript levels in the chick retina over a 24 h time period. *ZENK* transcript levels in the retina were significantly suppressed following 1 h of form-deprivation, and remained suppressed during the subsequent dark phase and the beginning of the following light phase, as compared to age-matched control values (ANOVA; F (2,46)=23.45, p<0.001) and contralateral control values (ANOVA; F (2,46)=13.34, p<0.01). The mean normalized expression is calculated from the efficiency (*E*) of the target genes to the power of its average CT value (*E*^CT^, target), divided by the efficiency (*E*) of the reference gene (β-actin) to the power of its average CT value (*E*^CT^, reference). Error bars represent SEM, n=5.

*ZENK* RNA transcript levels in the retina were significantly depressed following 1 h of form-deprivation (*t*-test; p<0.05; Figure 2) and remained depressed, compared to control values, over the entire time-course. The levels of *ZENK* in the contralateral control eye were not significantly affected during the development of FDM. Analysis of *ZENK* expression over a 12:12 h light–dark cycle indicated that once *ZENK* expression had been rapidly suppressed following 1h of form-deprivation, it did not recover during the subsequent dark phase, and was still suppressed at the beginning of the following light phase, compared to age-matched control values (MANOVA; F (2,46)=23.45, p<0.001) and contralateral control values (MANOVA; F (2,46)=13.34, p<0.01; Figure 3).

*ZENK* RNA transcript levels in the retina were significantly down-regulated 1h after fitting negative lenses (*t*-test; p<0.05; Figure 2). *ZENK* levels further declined after chicks were exposed to 1, 3, and 7 days of hyperopic defocus, as compared to that of contralateral control eyes (*t*-test; p<0.05, p<0.01, and p<0.05, respectively) and age-matched untreated eyes (*t*-test; p<0.05, p<0.01, and p<0.05, respectively), before showing a return toward baseline levels at day 10 of lens treatment (*t*-test; p=0.09).

### Changes in *PPG* RNA transcript expression during the development of FDM and LIM

The fitting of translucent diffusers or negative lenses significantly affected *PPG* RNA transcript levels in the experimental eye over time, as compared to both contralateral control values (MANOVA; F (2,86)=13.29, p<0.05 and F (2,86)=8.31, p<0.05, respectively) and age-matched untreated control values (MANOVA; F (2,86)=13.02, p<0.05 and F (2,86)=9.12, p<0.05, respectively). There was no significant difference over time in the expression of *PPG* transcript levels between contralateral control eyes, in either form-deprived animals or negative-lens-treated animals, and age-matched untreated values (MANOVA; F (2,86)=0.27, p=0.62 and F (2,86)=0.10, p=0.84, respectively).

**Figure 4:**
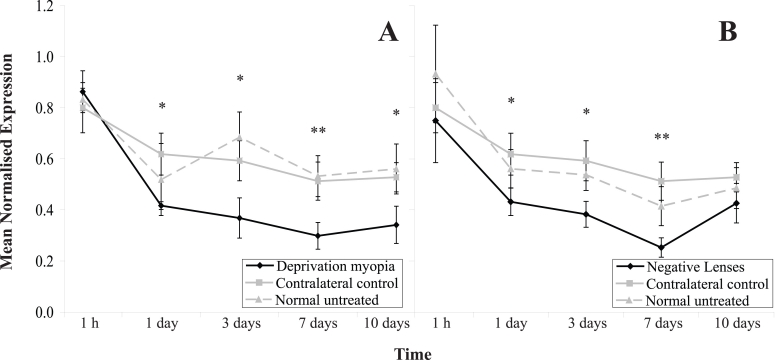
Changes in pre-proglucagon RNA transcript levels in treated and contralateral control retinas during periods of increased ocular growth induced by the fitment of translucent diffusers or negative lenses. Mean normalized expression of *PPG* RNA transcript levels from diffuser-treated (**A**) and negative-lens treated (**B**) eyes following 1 h, 1, 3, 7, and 10 days of treatment. The fitting of translucent diffusers or negative lenses significantly affected *PPG* RNA transcript levels in the experimental eye over time, as compared to both contralateral control values (MANOVA; F (2,86)=13.29, p<0.05 and F (2,86)=8.31, p<0.05, respectively) and age-matched untreated control values (MANOVA; F (2,86)=13.02, p<0.05 and F (2,86)=9.12, p<0.05, respectively). There was no significant difference in the expression of *PPG* transcript levels over time, between contralateral control eyes from either form-deprived animals or negative-lens-treated animals and age-matched untreated values (MANOVA; F (2,86)=0.27, p=0.62 and F (2,86)=0.10, p=0.84, respectively). The mean normalized expression is calculated from the efficiency (*E*) of the target genes to the power of its average CT value (*E*^CT^ target), divided by the efficiency (*E*) of the reference gene (β-*actin*) to the power of its average CT value (*E*^CT^ reference). Error bars represent SEM, n=9. (* p<0.05, ** p<0.01).

*PPG* RNA transcript levels in the retina were not significantly affected by 1h of form-deprivation (*t*-test; p=0.61; Figure 4). However, after one day of diffuser wear, *PPG* expression was significantly down-regulated (*t*-test, p<0.05), and remained so for the duration of the form-deprivation period (*t*-test; 3 days p<0.05, 7 days p<0.01, and 10 days p<0.05). The expression of retinal *PPG* transcripts in the contralateral control eye was not significantly different from that seen in age-matched control eyes at any point during the development of FDM (Figure 4).

During the development of LIM, *PPG* transcript levels in the retina showed a delayed but similar trend to that of *ZENK* levels (Figure 4). The expression of *PPG* was significantly down-regulated to below the levels seen in both contralateral control (*t*-test; p<0.05) and age-matched untreated eyes (*t*-test; p<0.05) after one day of lens wear. *PPG* expression further declined over the following six days of lens treatment (*t*-test; 3 days p<0.05 and 7 days p<0.01), before returning to control levels by day 10 of lens wear (*t*-test; p=0.39).

### Changes in *ZENK* expression in negative-lens-treated eyes following atropine or ADTN treatment

**Figure 5:**
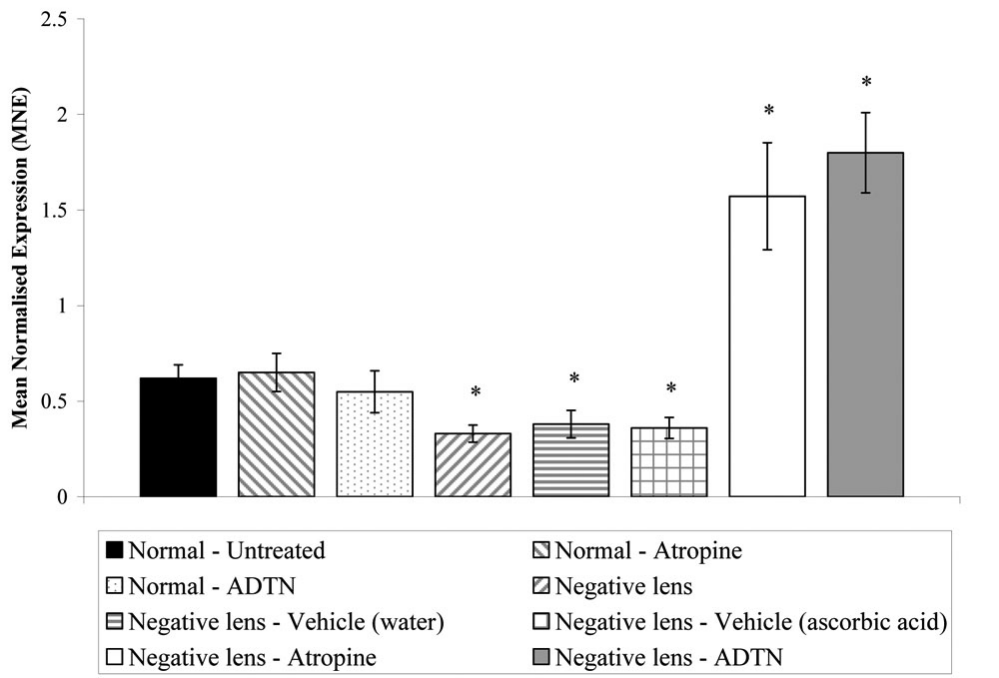
The effect of atropine and ADTN on *ZENK* transcript levels in the retina following 1 h of negative-lens wear. Negative-lens wear, for a period of 1h, induced significant down-regulation in *ZENK* transcript levels (ANOVA; F (4, 20)=4.24, p<0.05; *t*-test, p<0.05, respectively), as compared to normal untreated values, which was unaffected by the injection of either vehicle solution (distilled waster or ascorbic acid) immediately before lens fitting (*t*-test; p=0.7 and p=0.8, respectively). However, injection of atropine or ADTN immediately before the attachment of lenses induced significant upregulation in retinal *ZENK* expression above baseline levels (ANOVA; F (3, 14)=6.32, p<0.05; *t*-test, p<0.05, respectively). Atropine or ADTN did not affect retinal *ZENK* expression when injected into a normal untreated age-matched eye (ANOVA; F (3,14)=0.78, p=0.11; *t*-test, p=0.15, p=0.10, respectively). The mean normalized expression is calculated from the efficiency (*E*) of the target genes to the power of its average CT value (*E*^CT^ target), divided by the efficiency (*E*) of the reference gene (*Actb*) to the power of its average CT value (*E*^CT^ reference). Error bars represent SEM, n=6 (*p<0.05).

*ZENK* transcript levels were significantly down-regulated after 1h of negative-lens wear as compared to untreated control values (ANOVA; F (4,20)=4.24, p<0.05; *t*-test, p<0.05). This down-regulation was unaffected by injection of either vehicle solution (*t*-test; water, p=0.70; ascorbic acid, p=0.80; Figure 5). In contrast, the injection of the muscarinic cholinergic antagonist atropine or the dopamine agonist ADTN, immediately before the fitting of negative lenses, prevented the down-regulation in *ZENK* transcript levels observed after 1 h of negative-lens wear alone (ANOVA; F (3,14)=6.32, p<0.05; *t*-test, p<0.05), with *ZENK* levels rising above those seen in age-matched untreated eyes (ANOVA; F (3,14)=4.89, p<0.05; *t*-test; p<0.05). The injection of atropine or ADTN into control eyes not treated with negative lenses had no effect on the retinal expression of *ZENK* (ANOVA; F (3,14)=0.78, p=0.11; *t*-test; p=0.15 and p=0.10, respectively). Thus, *ZENK* RNA transcript levels in the retina were increased by atropine and ADTN only in circumstances that lead to suppression of excessive axial elongation.

## Discussion

### Changes in *ZENK* and *PPG* RNA transcript levels during periods of increased ocular growth

During periods of increased ocular growth induced by form-deprivation or negative lenses, *ZENK* transcript levels in the retina were rapidly suppressed, consistent with the changes observed at the peptide level by Fischer et al. [9] and at the RNA transcript level by Simon et al. [13], for negative-lens treatment. This down-regulation, however, was not transient, as *ZENK* expression remained down-regulated for much of the FDM and LIM treatment period.

The shape of the changes in *ZENK* transcript expression induced by imposed hyperopic defocus differed from that seen during development of FDM, which may be related to differences in the rate of ocular growth over the experimental period. Specifically, by day 7 of treatment, most chicks had fully compensated for the negative lenses, with a corresponding return of *ZENK* expression to baseline levels. In contrast, chicks that wore diffusers showed elevated levels of ocular growth over the entire ten days of treatment, with a corresponding continued reduction in *ZENK* expression.

The expression of IEGs such as *ZENK* are normally low in non-stimulated cells, but following external stimulation, their transcription is rapidly, and usually transiently, upregulated (for review see [24]). In contrast, the loss of form-vision or the attachment of negative lenses produced a rapid, yet prolonged, down-regulation in *ZENK* transcript levels. Morgan and Curran [25] have postulated that there are at least three broad categories of IEG response to external stimuli–the classical rapid and transient upregulation of IEGs; a delayed, yet prolonged, increase in gene expression; and a continuous tissue-specific expression of IEGs. The prolonged depression of *ZENK* in response to form-deprivation and negative lenses is therefore unusual. The prolonged depression could be explained by a renewed transient down-regulation in the *ZENK* transcript levels each day at the onset of light, and a new experience of form-deprivation or hyperopic defocus. A more detailed time-course over a light–dark cycle, however, reveals that the down-regulation of *ZENK* levels in the retina is continuous over subsequent periods of light and dark (Figure 3).

The prolonged down-regulation is also unlikely to be due to the slight reduction in light intensity (~0.4 log units) caused by the diffusers, since the fitting of negative lenses, which produces little change in light intensity, induces a similar suppression in *ZENK* levels in the retina. Overall, it appears that during periods of increased axial growth, induced by either the loss of form-vision or hyperopic defocus, *ZENK* RNA transcript levels in the retina are rapidly suppressed, and appear to remain down-regulated for the period of increased ocular growth. The return of *ZENK* expression to control levels at around the time when the imposed refractive error has been neutralized in the LIM paradigm may be particularly significant.

Changes in *ZENK* transcript expression were followed by a slower but similar change in retinal *PPG* transcript levels during both FDM and LIM. The initial increase in expression reported by Buck et al. [11] was not observed in this study. *PPG* expression in the retina returned to baseline levels following ten days of negative lens treatment, when the imposed refractive error had been largely compensated for. In contrast, following a similar period of form-deprivation, the rate of ocular growth would still have been elevated and the retinal expression of glucagon was still down-regulated. In general, changes in both *ZENK* and *PPG* transcript levels correlated well with the refractive changes observed during negative-lens compensation.

The pattern of changes observed is consistent with a pathway in which down-regulation of *ZENK* expression is followed by the down-regulation of the expression of *PPG* transcripts. Down-regulation of gene expression for the precursor of a peptide hormone/transmitter such as glucagon is most likely indicative of a reduced rate of synthesis of the peptide precursor, and of a decreased rate of glucagon release during the development of experimental myopia [26]. The ability of glucagon agonists to block experimental myopia [10,18] is consistent with such a pathway.

### Expression of *ZENK* in negative-lens-treated eyes following injection of atropine and ADTN

We have previously shown that the intravitreal injection of atropine or ADTN into the chick eye immediately before fitting diffusers prevents the down-regulation in *ZENK* RNA transcript levels normally observed after 1h, and in fact increased the levels of *ZENK* to above control levels [8]. Very similar results were obtained with the LIM paradigm. The ability of atropine and ADTN to upregulate *ZENK* expression is in line with the known induction of *ZENK* expression in the central nervous system by both muscarinic cholinergic antagonists [27,28] and dopaminergic agonists [29-33]. These results have several implications. They add to the evidence that upregulation of *ZENK* RNA or protein expression in the avian retina generally correlates with reduction in the rate of ocular growth, induced by the removal of diffusers from eyes subjected to prolonged form-deprivation or the wearing of positive lenses [9], or by the injection of atropine or ADTN into eyes developing myopia [8]. As with form-deprivation [8], the down-regulation of *ZENK* RNA transcript expression in the retina, induced by negative-lens wear, was extremely rapid on a biochemical timescale, with around a 40% reduction in levels of RNA transcripts within 1 h. Similarly, the reversal of this pattern of *ZENK* down-regulation by the injection of atropine or ADTN was also observed within 1 h, demonstrating that both compounds are capable of rapidly influencing one of the earliest known molecular changes observed in the retina during development of experimental myopia. Little is currently known about retinal interactions between cholinergic, dopaminergic, and glucagonergic cells in the avian retina, although Fischer et al. [34] have reported localization of the muscarinic-cholinergic receptor cm4 on all amacrine cells immunoreactive for tyrosine hydroxylase.

As previously discussed in relation to FDM [8], the differential effects of atropine and ADTN on *ZENK* RNA transcript levels in the retina and on longer-term eye growth, depending on whether or not lenses were fitted, suggest that a profound change is induced in the functioning of the retinal circuitry within the first hour, and probably within minutes, of exposure to form-deprivation or lens-induced hyperopic defocus. This suggests that the pathways involved in ‘normal’ eye growth, and those operating under conditions of ‘abnormal’ eye growth, are different in relation to the function of dopaminergic and cholinergic circuits in the retina within one hour of fitting the optical devices. Similarly, as previously discussed in relation to FDM, the speed of change at the retinal level in both FDM and LIM, in response to atropine and ADTN, suggests that these compounds are acting at retinal sites. Based on the current results we obviously cannot exclude other sites of action. Such sites include the choroid and RPE, which also show rapid changes during periods of altered ocular growth, particularly since there are also dopaminergic and muscarinic cholinergic pathways within these tissues [34-39], as well as the sclera, which is known to be affected directly by muscarinic antagonists [40,41]. But, if the effects of the drugs were exerted at non-retinal sites, there would need to be a rapid initiation of response outside the retina, as well as rapid transmission of this response to sites in the retina, to reverse in sign the retinal changes in levels of *ZENK* mRNA within 1 h. We therefore believe that the most plausible interpretation of our results is that atropine and ADTN act at retinal sites, but we recognize that the arguments are not conclusive. Work using a cholinotoxin and quisqualic acid to disrupt cholinergic pathways in the retina [42] has been interpreted as indicating that cholinergic amacrine cells are not critical for the control of eye growth. However, lesioning techniques are limited by the effectiveness of the toxins, and we believe that the current study provides stronger evidence in favor of retinal sites of action.

### Is the response of the eye to form-deprivation and hyperopic defocus modulated by similar underlying mechanisms?

Irrespective of the implications for sites of action of atropine and ADTN, a striking aspect of the pharmacological results, in conjunction with the time-course data discussed above, is the parallel between the molecular changes observed in response to lens-induced myopia and to form-deprivation. Although the morphological changes during the development of FDM and LIM are similar, animal studies have indicated that the underlying mechanisms may be different, due to differences in the time-course of changes in axial length [43], the effect of constant light [43] and brief periods of stroboscopic illumination [44], the effect of optic nerve sectioning [45], electroretinogram responses [46], and the effect of the dopaminergic toxin 6-hydroxydopamine (6-OHDA) [47]. However, more recent work has questioned these initial conclusions in relation to the effect of optic nerve sectioning [48], the effect of constant light [49], and the effect of 6-OHDA [50]. Further similarities are that both are blocked by the muscarinic antagonists atropine [51-53] and pirenzepine [54,55], the dopamine agonist apomorphine [52], the dopamine toxin 6-OHDA [47,50], reserpine (which depletes serotonin and dopamine vesicle stores) [56], the nitric oxide synthase inhibitor L-NAME [57,58], the glucagon agonist Lys17,18, Glu21-glucagon [10,59,60], and nitric oxide synthase (NOS) inhibitors [57,58]. In this paper, we show that the molecular changes in expression of *ZENK* and *PPG* are similar in FDM and LIM, and that both FDM and LIM appear to produce similar changes in retinal circuitry that increase the sensitivity of retinal circuits to dopaminergic agonists and muscarinic cholinergic antagonists within 1 h of the commencement of visual manipulation. There are, however, some remaining differences reported between these two paradigms [43,46], which require further investigation.

### Contralateral effects

Several studies have observed similar but less-pronounced changes in gene and peptide expression in the contralateral control eye, compared to those seen in the treated eye (see, for example [9,13,61-63]), and several possible mechanisms for these changes have been discussed [9]. In this study we did not find significant effects of the experimental manipulations in one eye on *ZENK* or *PPG* transcript levels in the contralateral eyes of treated animals at any point within the time-course investigated. However, it should be noted that there was some difference in the changes in refraction in contralateral eyes of FDM birds, namely a decline in hyperopia toward emmetropia, as compared to the changes in contralateral eyes of LIM birds where the initial hyperopic refraction was maintained. This issue also merits further systematic investigation, since contralateral changes in gene expression without changes in eye growth, or contralateral changes in eye growth without changes in gene expression, would raise serious questions about the causal relationships involved.

In summary, the results of this gene study support the idea that *ZENK* expression and the synthesis and release of glucagon may be important in the control of eye growth, and that changes in these two parameters may be related. Furthermore, injection of the non-specific muscarinic cholinergic antagonist atropine, and the non-specific dopaminergic agonist ADTN, prevents the rapid down-regulation in *ZENK* RNA transcript levels in the retina during the development of experimental myopia, induced by either negative-lens wear or form-deprivation, and in fact leads to marked increases in *ZENK* mRNA expression. Therefore, both atropine and ADTN appear to act on one of the earliest retinal signals produced during periods of increased ocular growth. The similar responses of retinal *ZENK* and *PPG* RNA transcript levels in FDM and LIM are consistent with substantial similarities in the cellular and molecular pathways underpinning these two forms of experimental myopia.
